# Global and Spatial Metabolomics of Individual Cells Using a Tapered Pneumatically Assisted nano-DESI Probe

**DOI:** 10.1021/jasms.3c00239

**Published:** 2023-10-13

**Authors:** Cátia Marques, Felix Friedrich, Liangwen Liu, Francesca Castoldi, Federico Pietrocola, Ingela Lanekoff

**Affiliations:** † Department of Chemistry—BMC, Uppsala University, 75123 Uppsala, Sweden; ‡ Department of Medical Cell Biology, Uppsala University, 75123 Uppsala, Sweden; § Department of Biosciences and Nutrition, Karolinska Institute, 14152 Huddinge, Sweden

## Abstract

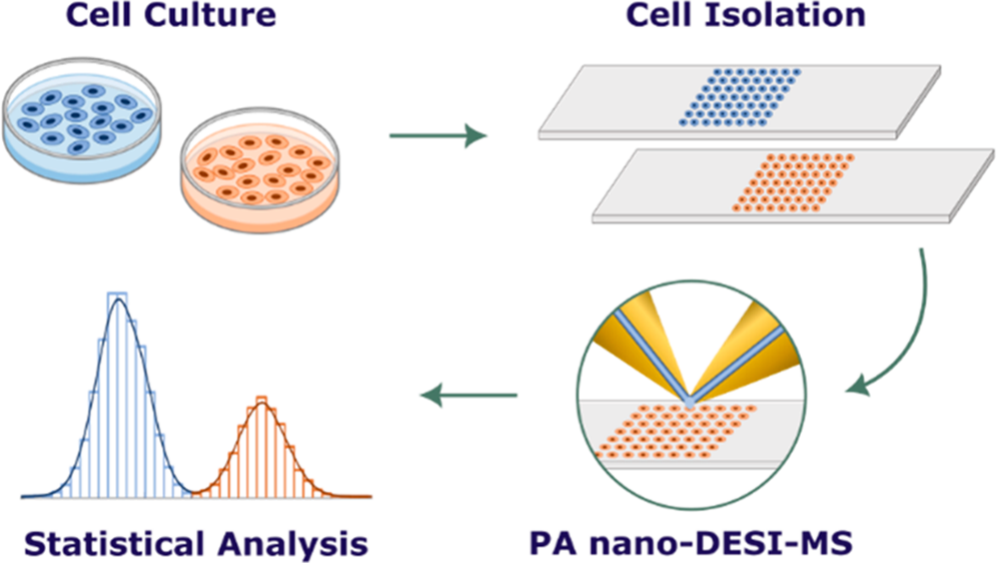

Single-cell metabolomics has the potential to reveal unique insights into intracellular mechanisms and biological processes. However, the detection of metabolites from individual cells is challenging due to their versatile chemical properties and concentrations. Here, we demonstrate a tapered probe for pneumatically assisted nanospray desorption electrospray ionization (PA nano-DESI) mass spectrometry that enables both chemical imaging of larger cells and global metabolomics of smaller 15 μm cells. Additionally, by depositing cells in predefined arrays, we show successful metabolomics from three individual INS-1 cells per minute, which enabled the acquisition of data from 479 individual cells. Several cells were used to optimize analytical conditions, and 93 or 97 cells were used to monitor metabolome alterations in INS-1 cells after exposure to a low or high glucose concentration, respectively. Our analytical approach offers insights into cellular heterogeneity and provides valuable information about cellular processes and responses in individual cells.

## Introduction

Chemical analysis of individual cells is essential to realize cellular heterogeneity, which is important to mitigate stress and improve resilience in a biological system.^1^ However, chemical alterations in individual cells can also be the origin of pathological processes, including aging and disease development.^1,2^ Efforts to study processes on a single-cell level have been achieved within all of the omics fields:^3^ genomics,^4^ transcriptomics,^5^ proteomics,^6^ and metabolomics.^7^ Although genomics and transcriptomics display the map for single-cell chemistry, the study of proteins and metabolites reveals the actual phenotype. With proteomics it is possible to study phenotypical alterations that occur from within hours to a few days.^8^ However, for changes within seconds to minutes, metabolomics provides a more comprehensive picture.^9^ Thus, analyzing the metabolome provides a snapshot of ongoing cellular processes.^10^

Single-cell metabolomics is challenging to achieve. This is partly due to the rapid turnover rate of metabolites but also the high complexity of the metabolome with diverse structures and wide concentration ranges within the picoliter-order cell volume.^9,11^ Although mass spectrometry (MS) is the main analytical tool used for single-cell metabolomics, it is used in combination with several different sampling and ionization strategies.^12−15^ The combined sampling and ionization strategies of individual cells include matrix-assisted laser desorption (MALDI)^16,17^ and secondary ion mass spectrometry (SIMS).^18,19^ For example, single-cell metabolomics using MALDI has revealed metabolic heterogeneity and states of stimulated dHepaRG hepatocytes, and different metabolic profiles related to inflammatory macrophages have been pinpointed by SIMS.^20,21^ Other sampling strategies for single-cell metabolomics are combined with electrospray ionization (ESI) MS.^22,23^ For example, electromigration combined with droplet-assisted ESI revealed the importance of isomeric lipids in drug-resistant HCC827 cells.^24^ These and other studies demonstrate the possibility and significance of performing metabolomics on individual cells.^12−15^

To achieve metabolomics from individual cells, different sampling strategies ensure that data are acquired from only one cell attached to a surface. For example, cell locations can be monitored using a microscope while simultaneously sampling individual cells.^25,26^ Alternatively, the exact location of individual cells can be pre-mapped by an image-guided software to efficiently sample only from specific locations.^17^ Another option is to sample the entire surface and to postprocess the data to only include cells that were sampled satisfactorily.^20^ All of these strategies were employed for individual cells grown on the substrate used for analysis. However, cells can also be placed in suspension and positioned in microwells or predefined arrays for easy targeting of individual cells.^27,28^ Generally, a predefined cell location would contribute to a higher throughput when analyzing the global metabolome.

Several sampling techniques have been used in combination with ESI MS.^7,12−15^ We have previously reported the applicability of nanospray desorption electrospray ionization (nano-DESI) for global metabolomics of individual cheek cells.^29^ Here, we are using pneumatically assisted (PA) nano-DESI that enhances metabolite signals in comparison to signals from lipids.^30^ Furthermore, by combining PA nano-DESI with a probe of tapered capillaries with a low inner diameter, we show cellular imaging of larger senescent IMR-90 cells and global metabolomics of individual INS-1 cells down to 15 μm in diameter. Furthermore, we show a throughput of three cells per minute by depositing cells in a cellular array and report quantitative comparison of differentially treated INS-1 cells. Overall, we show metabolomics optimization and exposure conditions from a total of 479 individual INS-1 cells.

## Experimental Section

### Chemicals and Prepared Solutions

Solvents were HLPC-grade methanol (MeOH) (Fisher Scientific), water from Milli-Q Plus, LC/MS-grade acetonitrile (Fisher Chemical), and formic acid (Merck). The standards used for quantitation were Cell Free Amino Acid Mixture-^15^N (Sigma-Aldrich), glucose-*d*_2_, γ-aminobutyric acid-*d*_2_ (GABA-*d*_2_), acetylcholine-*d*_9_, oleic acid-*d*_9_ (FA 18:1-*d*_9_), LPC 19:0, PC 11:0/11:0. Solvents for PA nano-DESI analysis were MeOH/H_2_O (9:1) with 0.1% formic acid or ACN/MeOH (9:1) with 0.1% formic acid (Table S1).

### Deposition of Standard (Glutamate) On Glass Slides

Using a CellenONE (Cellenion, Lyon, France), three solutions of 0.9, 1.9, and 38.83 mM glutamate in H_2_O were spotted on silanized glass slides in two arrays of 5 × 5 distanced by 800 μm while the distance between the spots within the array was 400 μm. The amount deposited was calculated from the measured drop volume (530, 500, and 500 pL, respectively) and the glutamate concentration.

### Cell Handling

IMR-90 (Figure S1) and INS-1 cells were cultured and washed as mentioned in the Supporting Information, respectively. After the final wash, the cells were kept in the buffer solution at a density of around 200 cells/μL until they were isolated. A CellenONE (Cellenion, Lyon, France) instrument equipped with a glass piezo capillary (P-20-CS) was utilized for single-cell isolation on an in-house silanized regular glass slide kept at 4 °C. All deposited INS-1 cells were isolated based on their diameter and elongation in order to exclude doublets or cell debris (Table S2). The cells were positioned in an array of 25 × 4 having a distance of 150 μm between the spots in both the *x*- and *y*-directions (Figure S2). After cell deposition, the slides were quickly frozen and stored at −80 °C until analysis.

### Sampling

We used the PA nano-DESI probe that has been previously described by Duncan et al.^30^ Briefly, two fused silica capillaries are placed at an angle similar to conventional nano-DESI.^31^ However, while the primary capillary supplies the solvent, the secondary is passed through a tee and nitrogen gas is used to establish a self-aspirating device based on the Venturi effect. The capillary dimensions used were 150:20 (OD:ID, Polymicro Technologies, L.L.C. Phoenix, AZ).The two meeting ends were tapered manually using an in-house constructed beveller, and the OD was measured in relation to the ID using a high-resolution Dinolite digital camera. Details on the parameters for different solvents and flow rates can be found in Table S3.

### Mass Spectrometric Parameters

A QExactive Basic instrument and an Orbitrap IQ-XTM Tribrid instrument (Thermo Fisher Scientific, Bremen, Germany) were used to acquire data in the positive mode between *m*/*z* 70–1000 using a mass resolution of 140k and 240k (*m*/Δ*m* at *m*/*z* 200), respectively. Detailed information about the experimental parameters used can be found in Table S3.

### Data Handling

Data from individual cells were extracted and quantified using an in-house-developed MATLAB (R2022b) script. Shortly, the data were imported using RawFileReader. NET dynamic link libraries were provided by ThermoFisher. After loading the data, signals acquired from cells were identified based on the signal-to-noise (S/N) ratio increase of endogenous ion species, e.g. choline at *m*/*z* 104.1070. These values were then extracted and filtered with an intensity threshold of 1E3, a detection frequency of 10%, and a 25% presence. Finally, all *m*/*z* values across experiments were aligned by using a 4.9 ppm cutoff. All ion images were constructed using the in-house-developed i2i.^32^ The histograms are made in RStudio, and the number of bins was automatically selected using the interquartile range based on the variance in the group (Freedman–Diaconis rule).^33^

## Results and Discussion

**Figure 1:**
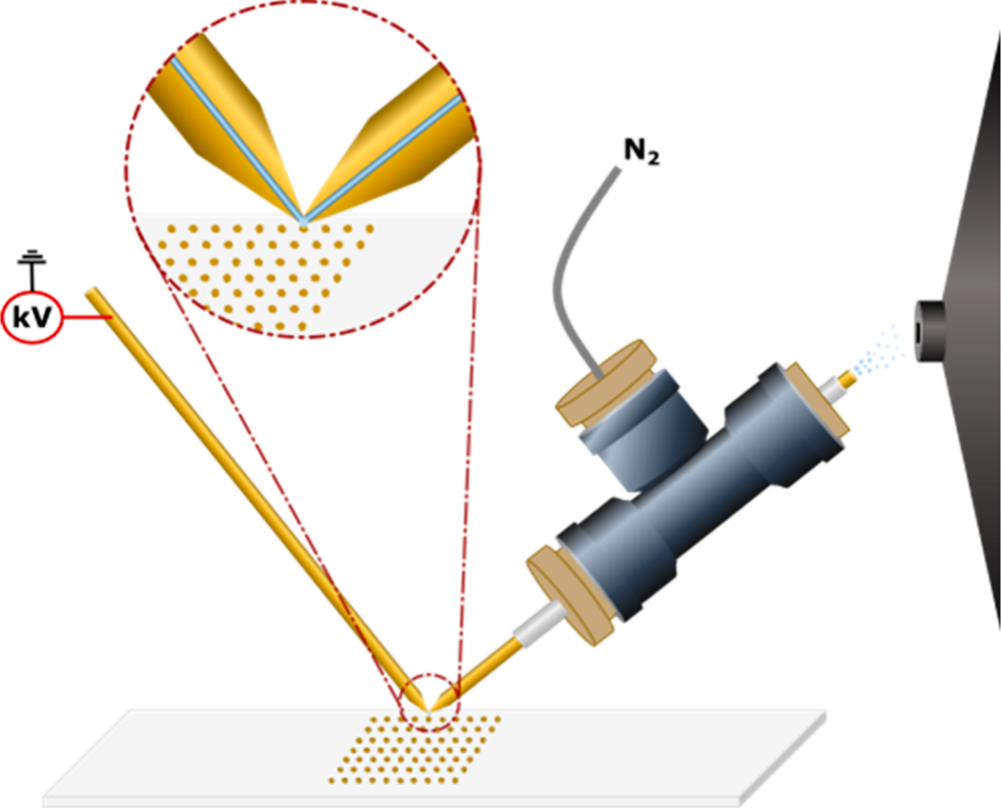
Schematic of the PA nano-DESI probe with tapered capillaries in the liquid bridge area (close up view).

**Figure 2:**
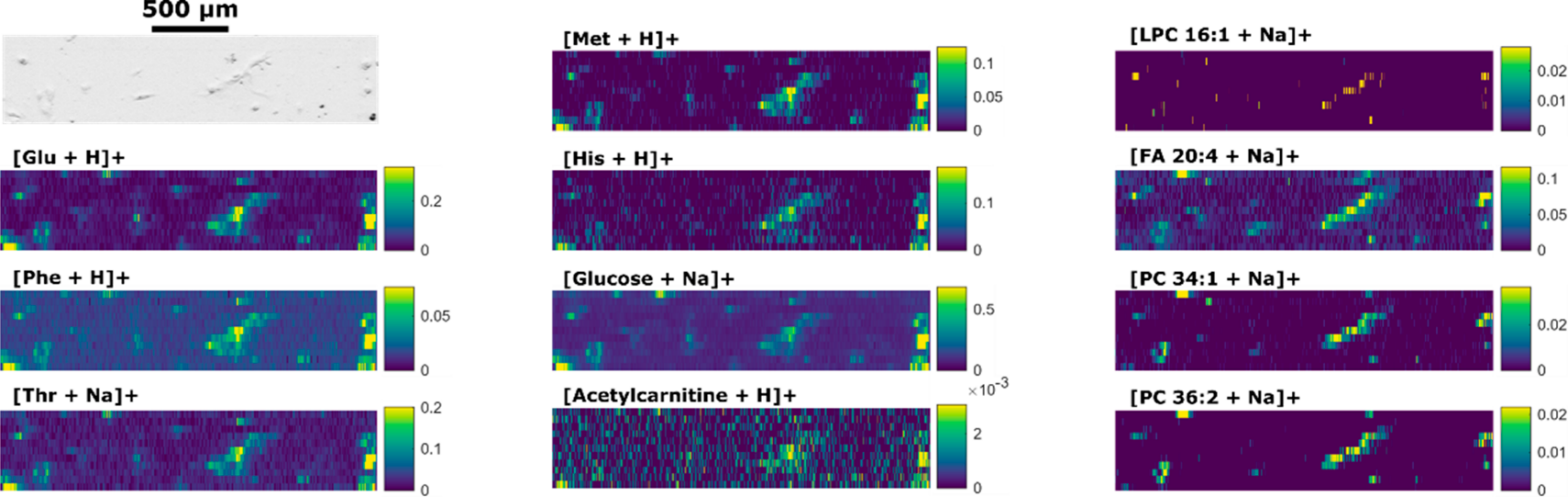
Ion images of metabolites and lipids detected from senescent single IMR-90 cells using PA nano-DESI with a tapered probe. The optical image shows the location of the cells cultured on the glass slide. All ion images are normalized to the respective internal standard, represented by the color map.

The tapered PA nano-DESI probe was set up with two fused silica capillaries miniaturized to an ∼23 μm outer diameter (Figure 1). The tapered probe significantly reduces the size of the liquid bridge between the two capillaries, and the retained inner diameter ensures an efficient transfer of desorbed material from the surface to the tip of the electrospray emitter. Similar to the nano-DESI probe, the tapered PA nano-DESI probe enabled mass spectrometry imaging (MSI) of cells cultured directly on glass slides.^29^Figure 2 shows ion images from metabolites and lipids that were acquired from senescent IMR-90 cells. Upon senescence, the cells increase in size, even up to ∼500 μm per cell (Figure S1).^34^ One of these large cells is highlighted in the optical image in Figure 2. With the tapered PA nano-DESI probe, it was possible to reduce the spacing of the lines in the *y*-direction to 50 μm without performing any oversampling. This allowed for six individual lines to be sampled from one large senescent cell, providing a comprehensive assessment of the metabolite distribution across the cell area. The results show that lipid (phosphatidylcholine (PC) and lysophosphatidylcholine (LPC)) species are evenly distributed over the large cell (Figures 2 and S3). In contrast, most amino acids show a heterogeneous distribution over the cell area. For example, glutamine, phenylalanine, and methionine show a higher abundance in the center of the cell (Figure 2). This suggests that the tapered PA nano-DESI probe enables spatial mapping of metabolites across the area of large cells.

Despite the promising results for cellular imaging of large cells, most mammalian cells are much smaller. As can be seen in Figure 2, both lipids and metabolites are detected also in the smaller senescent cells but at lower abundances (Figure S3). During imaging, the sample is constantly moved under the probe at 5 μm/s. Thus, the chemical material may not be exhaustively sampled from the individual cells.

A strategy different from the imaging mode could provide more chemical information from each single cell. Specifically, the touch-down mode consists of placing the probe on top of a cell until the signal has fully decayed, then the probe is lifted and the next cell is targeted. However, this requires that the location of small cells on the glass slide is known, since it is not possible to visualize manually.

**Figure 3:**
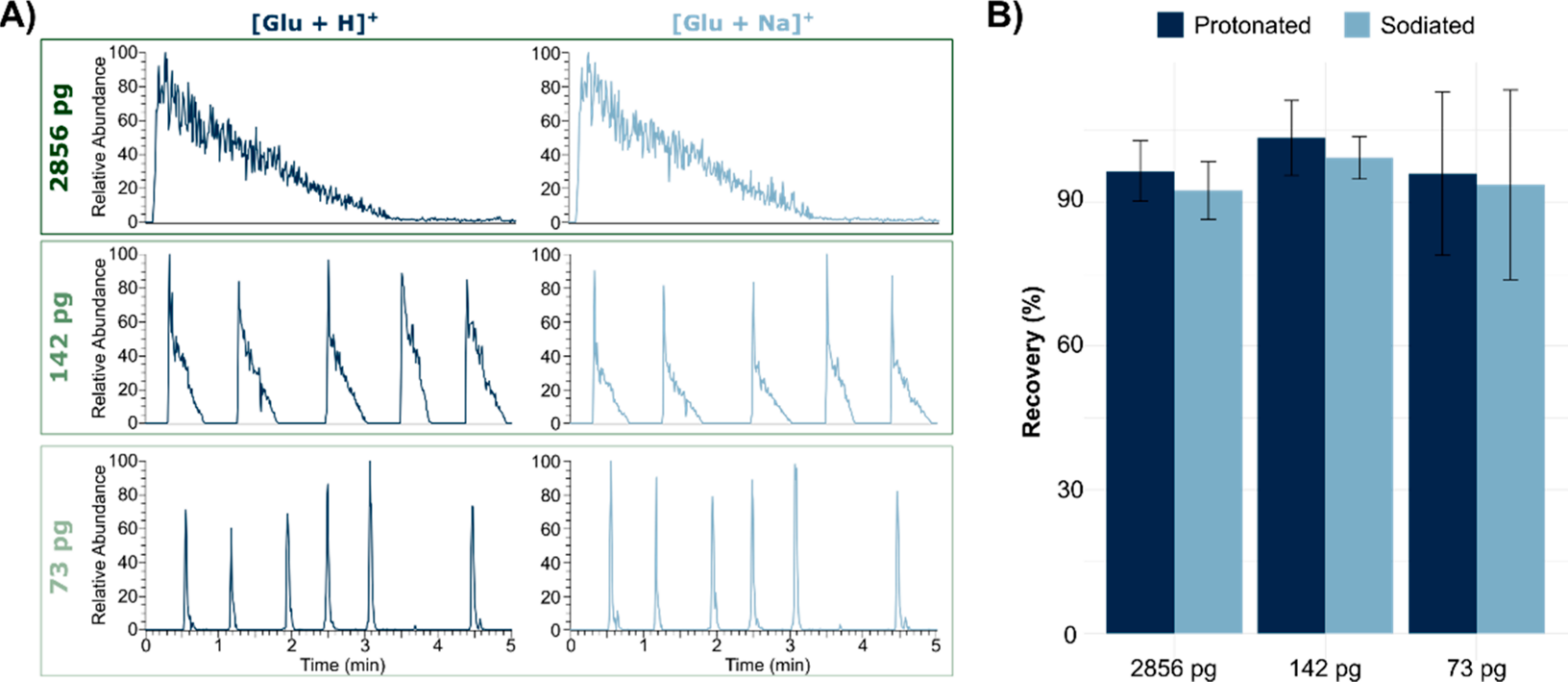
Touch-down mode of glutamate spotted on silanized glass slides using the tapered PA nano-DESI probe. (A) Chronograms of spots with different amounts of signal decay to baseline. (B) Estimated recovery between spotted and detected amounts. Error bars represent one standard deviation of 20, 29, and 14 spots of 2856, 142, and 73 pg of glutamate, respectively.

To evaluate the possibility of exhaustive extraction with the touch-down mode, known amounts of glutamate standard were spotted in known locations on silanized glass slides. Specifically, the standard drops were spotted in arrays and distanced by 400 μm in both the *x*- and *y*-directions (Figure S4). Then, the tapered PA nano-DESI probe was positioned on each spot, using the touch-down mode, until the signal had fully decayed and the material was presumed to be exhaustively extracted and analyzed. The result from the deposition of three different amounts of glutamate is shown in Figure 3A. The figure clearly shows that the signal decays faster for the spots with less material and that there is no carryover between the individual touchdowns. To further evaluate the correlation between the spotted amount and the detected amount (*n*_detected_, mol) we used the ^15^N-glutamate that was included in the PA nano-DESI solvent as an internal standard (IS) and calculated the detected amount according to the formula in eq 1.^35,36^
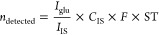
1In eq 1, the intensity ratio of the spotted glutamate standard (*I*_glu_) to the deuterated ^15^N-glutamate IS (*I*_IS_) is multiplied by the concentration of the IS (C_IS_, μM), the flow rate (F, μL/s), and the total scan time (ST, s), as previously described.^36^ The resulting correlation between spotted and detected amounts, as shown in Figure 3B, indicates that glutamate is indeed exhaustively extracted from the analyzed spots. Additionally, the results suggest that there is a higher variability in extraction and detection from the spots containing a lower amount of material. This could be a combination of a higher uncertainty in spotting the standard, extracting the standard, and detecting the analyte when dealing with minute amounts. Overall, the touchdown approach is promising both for quantitation and for higher throughput of single-cell metabolomics.

**Figure 4:**
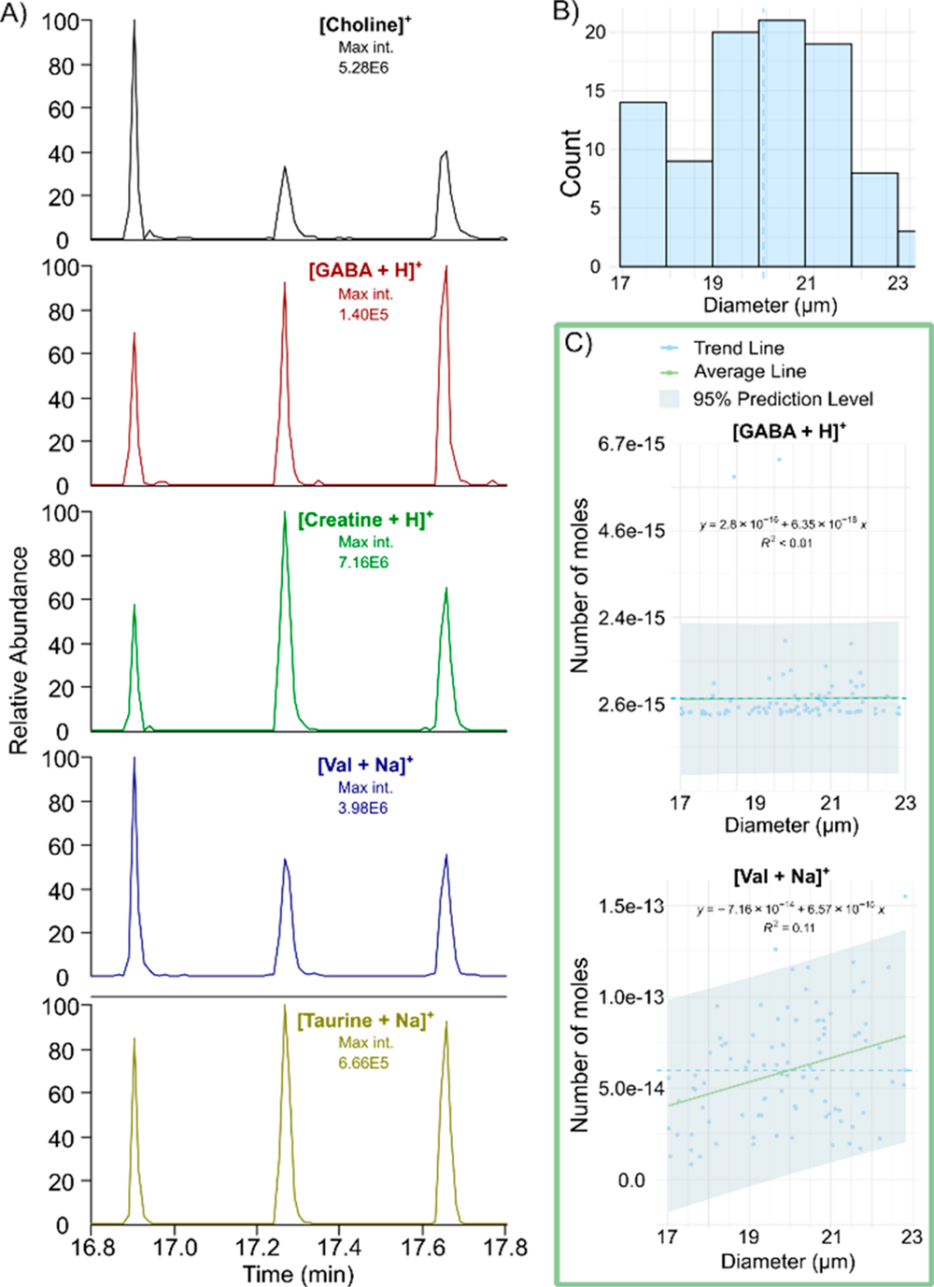
Analysis of individual INS-1 cells. (A) Extracted ion chronograms for several endogenous metabolites in three individual INS-1 cells analyzed after each other in touch-down mode. (B) Histogram showing the distribution of the cell diameter (17–23 μm) for 93 analyzed INS-1 cells. (C) Correlation between cell diameter and metabolite concentration shows that values fall within the 95% confidence interval (gray shade).

Contrarily to large senescent IRM-90 cells, individual INS-1 cells are smaller with diameter sizes from 15 to 24 μm (Table S2), which makes the chemical material per cell highly limited. Nevertheless, the tapered PA nano-DESI probe successfully extracts material from individual INS-1 cells for good signal detection of both metabolites and lipids (Figure S5). In an experiment, individual cells were deposited on silanized glass slides at known locations and flash frozen prior to analysis with the touch-down mode (Figure S2). The touch-down approach shows that chemical material from individual INS-1 cells is readily analyzed with a throughput of up to 3 cells/min (Figure 4A). In another experiment, the diameter of 93 deposited cells was selected to be between 17 and 23 μm (Figure 4B). Following analysis, the size of the cell was correlated with the amount of detected GABA and valine, as determined by eq 1 and the internal standards deuterated GABA and ^15^N-valine, respectively. The results displayed in Figure 4C show no distinct correlation between the detected analyte and the cell diameter. Thus, we decided that no corrections are applied in the data analysis that would account for differences in the cell diameter of individual INS-1 cells ranging between 17 and 23 μm. Overall, the tapered PA nano-DESI probe easily detects a large number of metabolites and lipids from individual INS-1 cells despite their small size.

**Figure 5:**
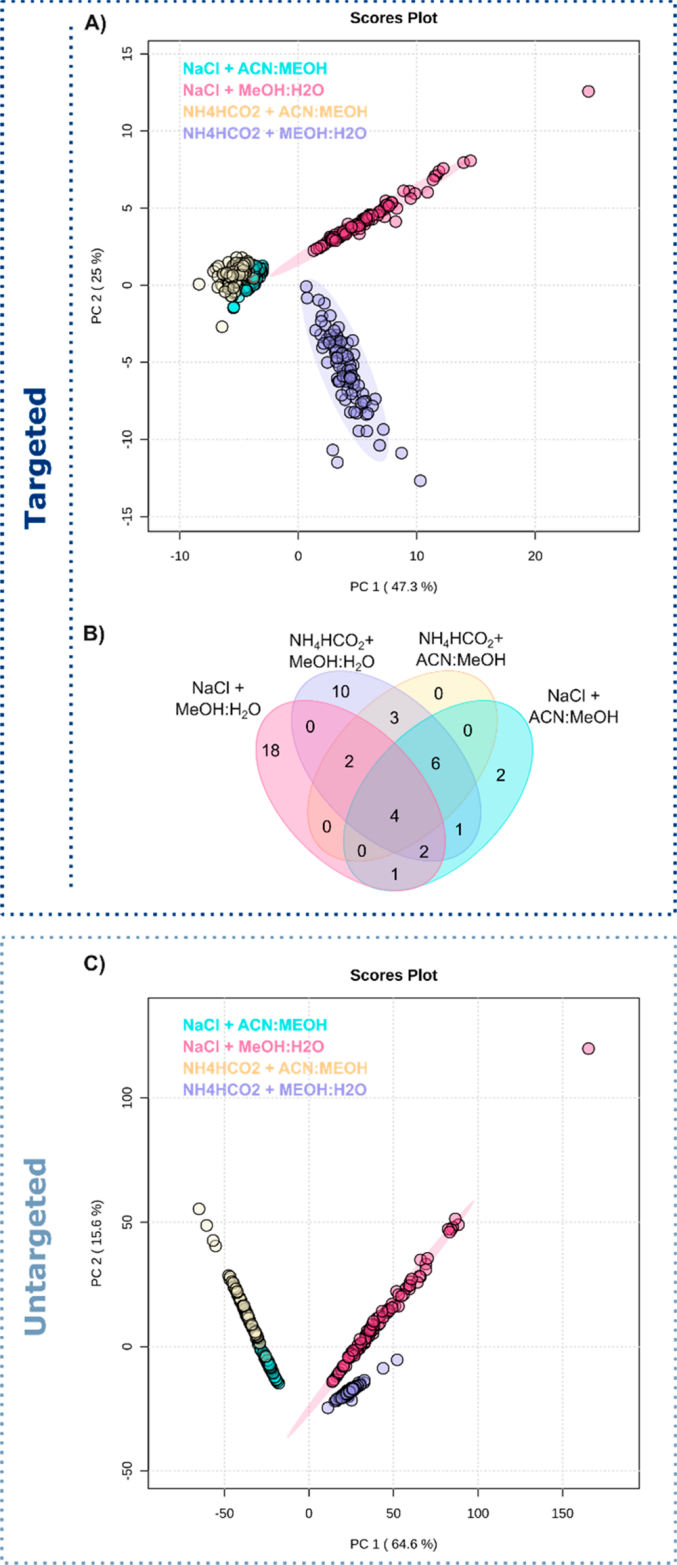
Optimization of four conditions for cell handling and analysis. (A) Principal component analysis of putatively assigned features. (B) Venn diagram of putatively assigned features. (C) Principal component analysis of all detected *m*/*z* values. A total of 382 individual INS-1 cells were used for the analysis.

To ensure the efficient detection of chemical material from individual INS-1 cells, the buffer used for cell deposition on slides and the solvent used for analysis were optimized. The selection of the washing buffer is important for maintaining cell homeostasis and may influence the total chemical matrix that is analyzed from the cell, including contamination.^37^ Here, we have evaluated the effect of two solutions for cell deposition, a saline solution (0.9% sodium chloride, NaCl) and 140 mM ammonium formate (NH_4_HCO_2_). Additionally, we have compared two different nano-DESI solvents, MeOH/H_2_O (9:1 v/v), and ACN/MeOH (9:1 v/v), both with 0.1% formic acid, and separately optimized instrumental settings (Table S1). Both solvents included internal standards in the form of labeled and nonendogenous analytes compounds (amino acids, lipids, etc.) to enable targeted quantitation. In total, 382 individual INS-1 cells were analyzed to test four combinations for single-cell metabolomics: NaCl with MeOH/H_2_O (91 cells), NaCl with ACN/MeOH (100 cells), NH_4_HCO_2_ with MeOH/H_2_O (93 cells), and NH_4_HCO_2_ with ACN/MeOH (98 cells). Principal component analysis (PCA) of putatively annotated features (targeted) from individual INS-1 cells shows a clear separation between different groups, although the solvents with ACN cluster together (Figure 5A, Table S4). A closer look into the number of annotated features shows that more analytes (>150%) are detected with the nano-DESI solvent MeOH/H_2_O compared to ACN/MeOH (Figure 5B and Table 1). This may be a result of both the extraction efficiency and the ionization efficiency, with the overall efficiency being higher for the MeOH/H_2_O solvent. This may also be the reason for the ACN containing solvents clustering together in Figure 5A. For the comparison of buffers for cell handling prior to analysis, a PCA plot for all detected *m*/*z* values (untargeted) using the different conditions shows a similar separation of these groups (Figure 5C). The higher separation for the solvents when using NaCl in both targeted and untargeted PCA is likely due to the difference in the solubility of NaCl in the solvents. Specifically, NaCl has a much higher solubility in MeOH (14.9 g/L) compared to ACN (0.003 g/L).^38^ The increased solubility of NaCl in MeOH was also observed in the mass spectrum as NaCl clusters, which may induce ion suppression for analytes, contaminate the mass spectrum, and risk overlapping peaks (Figure S6). Overall, the combination of washing with NH_4_HCO_2_ and analyzing with MeOH/H_2_O was found to be optimal due to fewer NaCl clusters and a higher number of features detected from single INS-1 cells.

**Table 1:** Number of Putatively Assigned Metabolites and Detected*m/z* Values in Each Buffer + Solvent Combination^a^

	MeOH/H_2_O		ACN/MeOH	
	assigned	features	assigned	features
NaCl	27	1713	16	1339
NH_4_HCO_2_	28	1984	15	1219

a A total of 383 individual INS-1 cells were used for the analysis.

**Figure 6:**
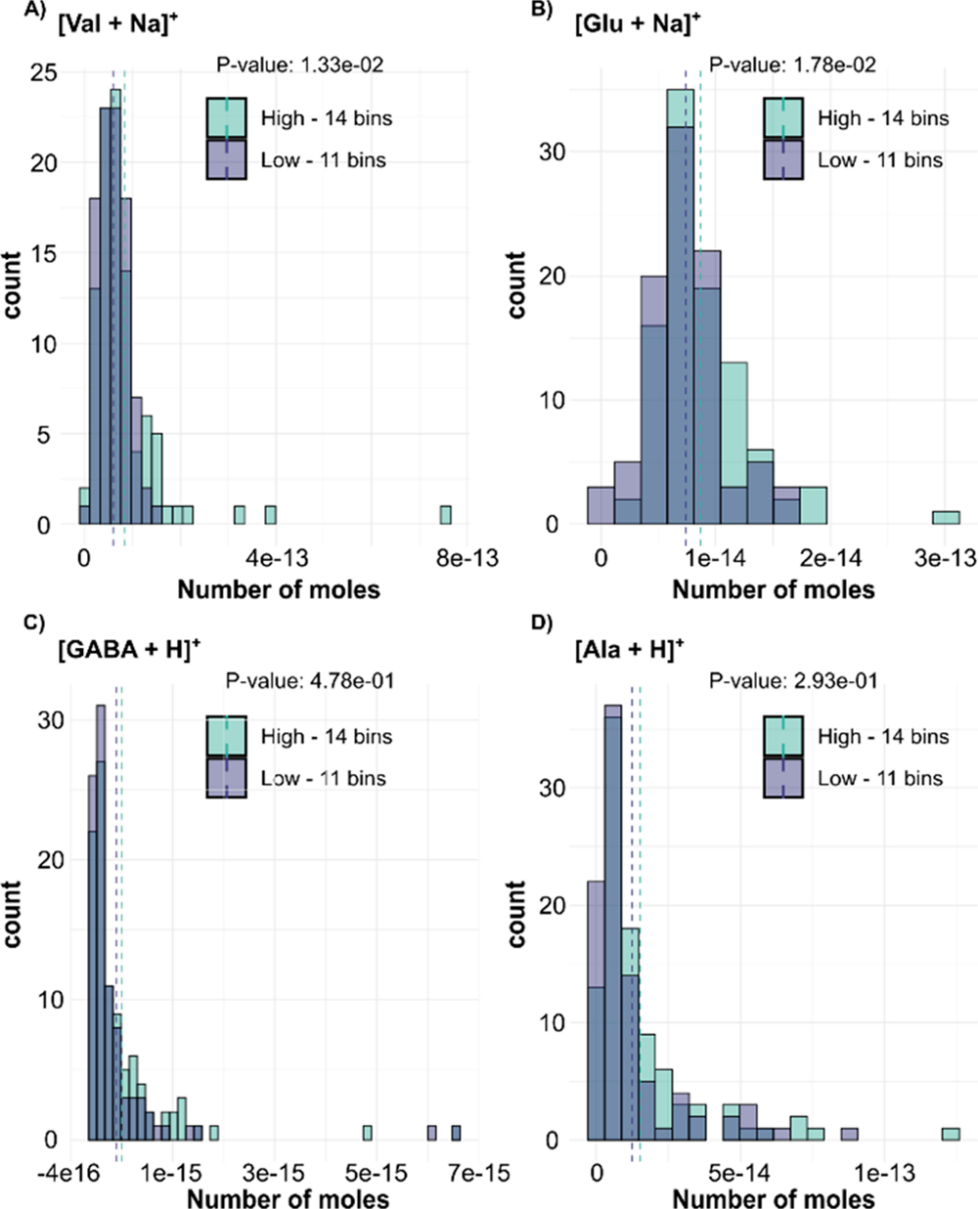
Histograms showing the distribution of selected endogenous metabolites from individual INS-1 cells exposed to a low (1 mM, purple) or high (20 mM, green) glucose concentration: (A) [Val + Na]^+^, (B) [Glu + Na]^+^, (C) [GABA + H]^+^, and (D) [Ala + H]^+^. The results shown for [Val + Na]^+^ and [Glu + Na]^+^ are significantly different (*p* < 0.05).

We and others have previously reported metabolome alterations of INS-1 cells treated with low and high glucose prior to homogenization and bulk analysis.^39−42^ Specifically, with bulk analysis we showed a large number of metabolites that were significantly altered between the two conditions using the direct infusion probe.^43^ Here, we investigate if metabolome alterations can also be detected in individual INS-1 cells under the same treatment conditions. Using the established optimized conditions, the tapered PA nano-DESI probe was used in touch-down mode to analyze 93 and 97 INS-1 cells treated with 1 or 20 mM glucose for 15 min, respectively. Figure 6 shows the resulting histograms for four small metabolites, *i.e.*, valine, glutamate, GABA, and alanine, in the two conditions. The histograms display the distribution of the number of cells in each bin within a similar detected number of moles (using eq 1). The histograms show a wide range of concentrations of the detected metabolites but no distinct subpopulations within either treatment group (Figure 6). However, the results show significant differences between the two treatment groups for both valine and glutamate according to a two-tailed unpaired heteroscedastic Student’s *t* test analysis, suggesting that there is indeed a heterogeneity between individual cells exposed to different treatments. This information is crucial for realizing cellular responses and variabilities within the seemingly identical population of cells, and future replicate experiments will further investigate these metabolome alterations. It is important to highlight that this difference within the groups is not due to the actual sampling time of the individual cells (Figure S7). Overall, differentiation between the two treatment populations was achieved, and several metabolites could be detected from single INS-1 cells using the PA nano-DESI tapered probe using a total of 190 cells.

## Conclusions

We present the tapered PA nano-DESI probe with an optimized buffer and solvent for the analysis of individual cells. Results from imaging indicate heterogeneous distributions of metabolites and uniform distributions of lipids. Despite the possibility for imaging, we show higher throughput for metabolomics of individual cells spotted in an array and analyzed using the touch-down mode with the analysis of 3 cells/min. Quantitative data suggest no significant bias to the cell diameter between 17 and 23 μm. Finally, we used the established method and reported differences in metabolite concentrations of INS-1 cells exposed to a high or low glucose concentration.

## Supporting Information Available

IMR-90 and INS-1 cell culture and handling, internal standards concentrations, staining of cells to evaluate senescence, parameters for cell isolation with CellenONE, array of isolated cells, MS parameters, ion images of metabolites and lipids detected on single cells, array of Glu spotting with CellenONE, mass spectrum from an individual single cell, putative assignment of detected *m*/*z*, comparison of mass spectra when different cell washing buffers were used, and distribution of estimated moles for detected endogenous metabolites from single cells (PDF)
