# Supplementary material for: Global and Spatial Metabolomics of Individual Cells Using a Tapered Pneumatically Assisted nano-DESI Probe
Source: J Am Soc Mass Spectrom. 2023 Oct 13;34(11):2518–24. doi: 10.1021/jasms.3c00239 (PMC10623638; doi:10.1021/jasms.3c00239)
Supplement: Supplementary file 1 — js3c00239_si_001.pdf [file js3c00239_si_001.pdf]

Supporting Information

Global and spatial metabolomics of individual cells using a tapered pneumatically assisted nano-DESI probe

Cátia Marques[1], Felix Friedrich[1], Liangwen Liu[2], Francesca Castoldi[3], Federico Pietrocola[3], Ingela Lanekoff[1]*

[1] Department of Chemistry – BMC, Uppsala University, 75123 Uppsala, Sweden
[2] Department of Medical Cell Biology, Uppsala University, 75123 Uppsala, Sweden
[3] Department of Biosciences and Nutrition, Karolinska Institute, 14152 Huddinge, Sweden

Corresponding author:
Prof. Ingela Lanekoff
Ingela.Lanekoff@kemi.uu.se
Dept. of Chemistry-BMC (576)
Uppsala University
751 23 Uppsala
Sweden

Experimental details

IMR-90 cell culture and handling

IMR-90 (human lung fibroblasts) cells were obtained from ATCC. IMR-90 cells were maintained in standard DMEM, supplemented with 10% heat-inactivated fetal bovine serum (FBS; Gibco), Glutamine (2 mM; Gibco), 1% Non-Essential acids (1%; Gibco) and 1% antibiotics (penicillin/streptomycin 100 U/mL; Gibco). Cells were maintained in a humidified incubator at 37°C and 5% CO2. Cells were tested monthly for Mycoplasma contamination using Mycoalert™ Mycoplasma Detection Kit (Lonza™ LT07-318), and only negative cells were used for experiment. For analysis of individual cells $1x10^4$ cells were seeded on Lab-Tek II Chamber slide. Senescence was induced by treatment with the DNA-damaging agent doxorubicin (150 nmol/L; Sigma, #D1515) for 10 days. Induction of senescence was confirmed by detection of Senescence Associated Beta Galactosidase enzymatic assay (Figure S1), as previously described.[1] Ten days after treatment, the slide chamber was removed, cells were washed twice with deionized water and slides were snap-frozen in liquid nitrogen.

INS-1 cell culture and handling

Rat insulinoma cell line INS-1 clone 832/13 cells were maintained in RPMI 1640 (Invitrogen) containing 10 mM glucose and supplemented with 10% fetal bovine serum, penicillin (100 μg/mL), streptomycin (100 μg/mL), Napyruvate (1 mM), L-glutamine (2 mM), and β-mercaptoethanol (50 μM). The cells were kept at 37 °C in a humid atmosphere containing 5% CO2. For analysis of individual cells, approximately 1 million cells were counted and grown onto a T-25 cell culture flask. After ~24 h, flasks of cells were rinsed and incubated with 3mM glucose for 1h at 37 °C without CO2. Following, cells were either exposured to 1 mM glucose or 20 mM glucose for 15 min at 37 °C without CO2. The cells were washed with PBS and detached by adding trypsin for 3 min. Culture medium was added and the cell suspension was centrifuged at 900 rpm for 3 min. The medium was subsequently removed and the cells were washed three times with a buffer solution – either saline solution or 140 mM ammonium formate. The cells were kept in suspension for up to 30 minutes during sorting onto slides. These 30 minutes are the difference in time compared to our previous protocol.[2]

Table S1. Concentrations (μM) of standard solutions used in the MeOH:H_2O extraction solvent.

	Concentration (μM)
LPC 19:0	0.8
PC 11:0/11:0	0.9
Acetylcholine-d_9	0.1
Glucose-d_2	0.9
GABA-d_2	1.5
Arachidonic-d_8	0.7
FA 18:1-d_9	0.3
Glutamate-d_3	0.7
MG 19:2	0.8
DG 26:0	0.8
Malic and succinic acid $^{13}C4$	0.7
carnitine-d_3	0.3
C18-carnitine-d_3	0.4
Alanine-^{15}N	5.9
Arginine-^{15}N	1.2
Asparagine-^{15}N	6.6
Aspartic acid-^{15}N	6.6
Cysteine-^{15}N	1.6
Glutamic acid-^{15}N	3.8
Glutamine-^{15}N	1.6
Glycine-^{15}N	4.5
Histidine-^{15}N	0.4
Isoleucine-^{15}N	2.2
Leucine-^{15}N	3.5
Lysine-^{15}N	1.3
Methionine-^{15}N	0.6
Phenylalanine-^{15}N	1.6
Proline-^{15}N	1.6
Serine-^{15}N	2.3
Threonine-^{15}N	2.6
Tryptophan-^{15}N	1.6
Tyrosine-^{15}N	0.8
Valine-^{15}N	2.8

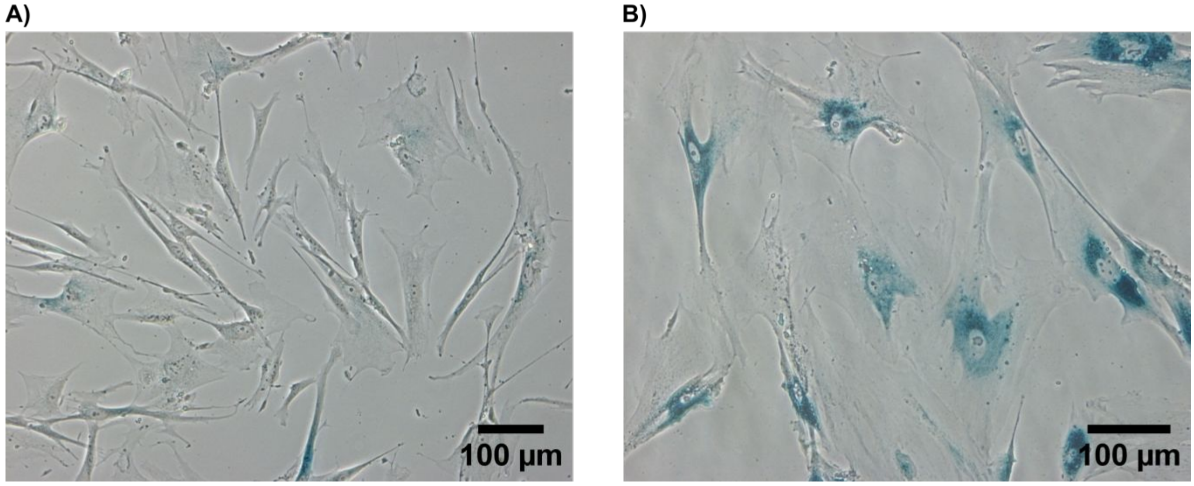

Figure S1. Senescence-associated beta-galactosidase staining of IMR90 cells, control (A) or treated (B) with doxorubicin to induce senescence. Representative images. Scale bar: 100 micrometers.

Table S2. Parameters used to isolate INS-1 cells with the CellenONE

	NaCl		NH_4HCO_2	
	**Low glucose**	**Low glucose**	**Low glucose**	**High glucose**
Extraction solvent	MeOH:H_2O	ACN:MeOH	MeOH:H_2O and ACN:MeOH	MeOH:H_2O
Diameter range (µm)	17-24	15-24	17-23	
Max Elongation	1.4	1.6	1.6	1.5
Nozzle current (V)	81	44	45	
Pulse shape	50 *µs*	ULV01	ULV01	ULV01
Humidity (%)	15	18	18	18
Grid specifications				
Spots across X	10	25	25	25
Distance between spots across X (µm)	250	150	150	150
Spots across Y	10	4	4	4
Distance between spots across Y (µm)	250	150	150	150

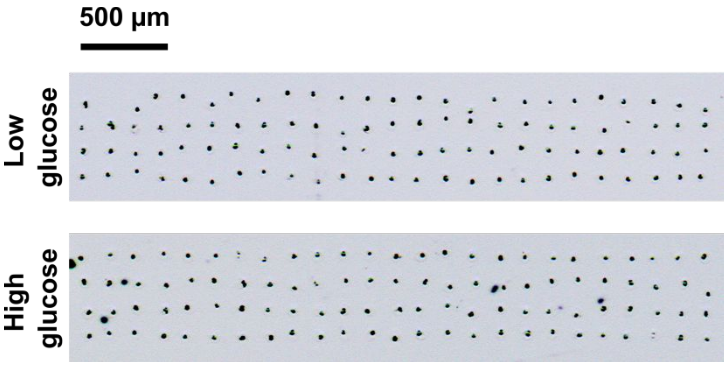

Figure S2. Slide scanner images of INS-1 cells exposed to low and high glucose spotted in a matrix of 25 x 4 using the cellenONE with a distance of 150 μm between spots. The occasional misalignment of the cells occurs during spotting and is adjusted for during analysis for spots containing individual cells.

Table S3. Mass spectrometer parameters used in the different experiments.

	QExactive Basic		Orbitrap™ IQ-X™ Tribrid™		
Cell type	**IMR-90**	**INS-1**	**INS-1**	**INS-1**	**INS-1**
Buffer/washing step	deionized water	NaCl	NaCl	NH_4HCO_2	NH_4HCO_2
Extraction solvent	MeOH:H_2O	MeOH:H_2O	ACN:MeOH	MeOH:H_2O	ACN:MeOH
Flow rate (µL/min)	0.15	0.12	0.42	0.15	0.42
Voltage (kV)	3.5	3.5	4.25	3.6	4.25
N_2 pressure (bar)	6	6	5	5	5
Capillary Temperature (°C)	300	300	315	315	315
Maximum injection time (ms)	300	300	300	300	300

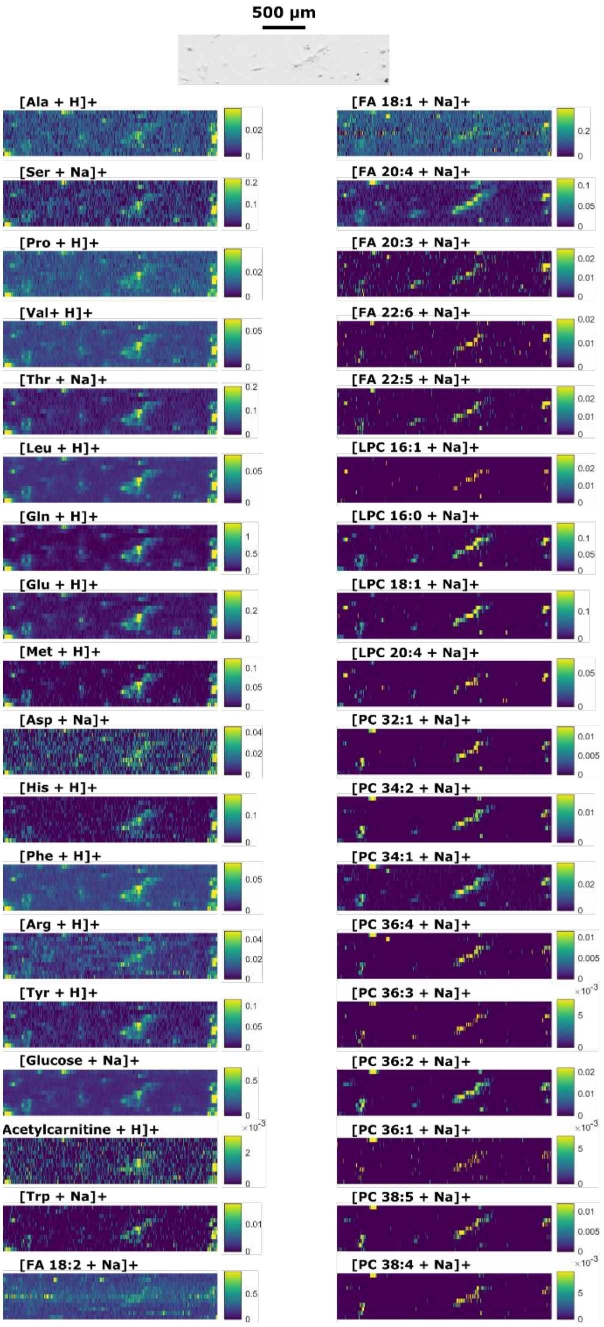

Figure S3. Distribution of metabolites and lipids over the large senescent IMR-90 cell. All are normalized to their respective internal standard.

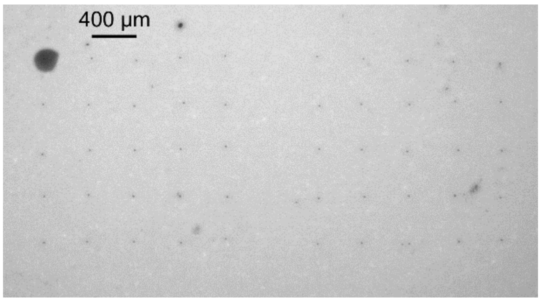

Figure S4. Image of Glu spotting in two arrays of 5 x 5 distanced by 800 μm. the distance between the spots within the array is 400 μm.

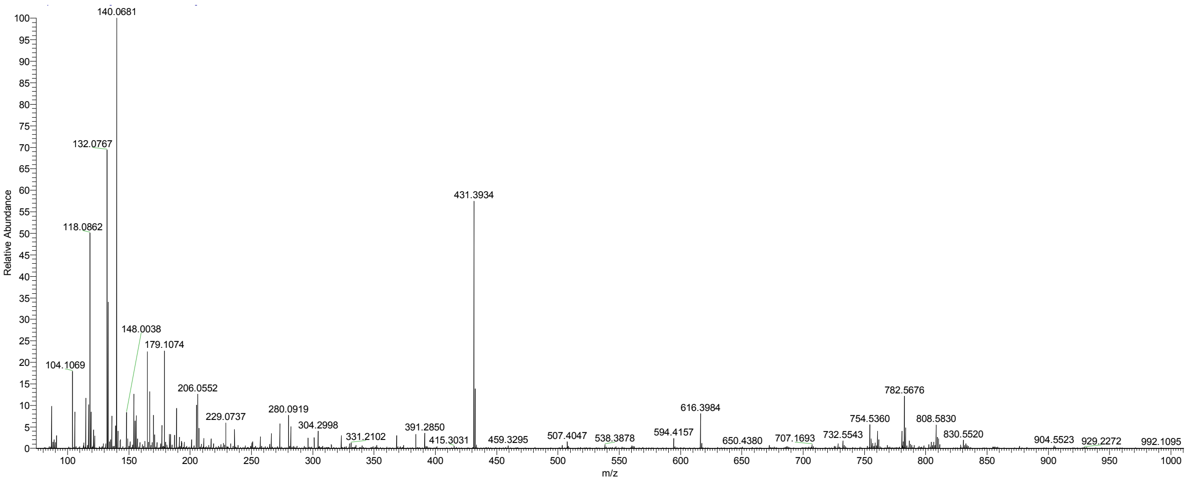

Figure S5. A mass spectrum from an individual INS-1 cell acquired with the tapered PA nano-DESI probe. The cell was extracted to completion, as measured by the decay in signal, using the touch-down procedure. The 9 spectra are averaged and background subtracted with scans acquired after the cell was analyzed using XCalibur.

Table S4. Putatively assigned metabolites and lipids used in evaluating the different buffer and solvent conditions. The m/z values in green were detected in INS-1 single cells with treated and extracted with NH_4HCO_2 and $MeOH:H_2O$.s

Analyte	***[M+H]⁺***	***[M+Na]⁺***	***[M+K]⁺***
20α,22R-Dihydroxycholesterol	419.3520	441.3339	457.3079
Ala	90.0550	112.0369	128.0108
Asp	134.0448	156.0267	172.0007
C2 carnitine	204.1230	226.1050	242.0789
Creatine	132.0768	154.0587	170.0326
Creatinine	114.0662	136.0481	152.0221
GABA	104.0706	126.0526	142.0265
Glu	148.0604	170.0424	186.0163
Glycerylphosphorylethanolamine	216.0632	238.0451	254.0190
GPC	258.1101	280.0920	296.0660
Hypoxanthine	137.0458	159.0277	175.0017
LPE 20:0	510.3554	532.3374	548.3113
Oxoproline	130.0499	152.0318	168.0058
PC 32:0	734.5694	756.5514	772.5253
PC 32:1	732.5538	754.5357	770.5097
PC 32:2	730.5381	752.5201	768.4940
PC 34:1	760.5851	782.5670	798.5410
PC 34:2	758.5694	780.5514	796.5253
PC 36:1	788.6164	810.5983	826.5723
PC 36:2	786.6007	808.5827	824.5566
Pro	116.0706	138.0526	154.0265
Taurine	126.0219	148.0039	163.9778
TG 50:3	829.7280	851.7099	867.6838
Thr	120.0655	142.0475	158.0214
Val	118.0863	140.0682	156.0421
		[M]⁺	
Acetylcholine		146.1176	
Choline		104.1070	
Phosphocholine		184.0733	

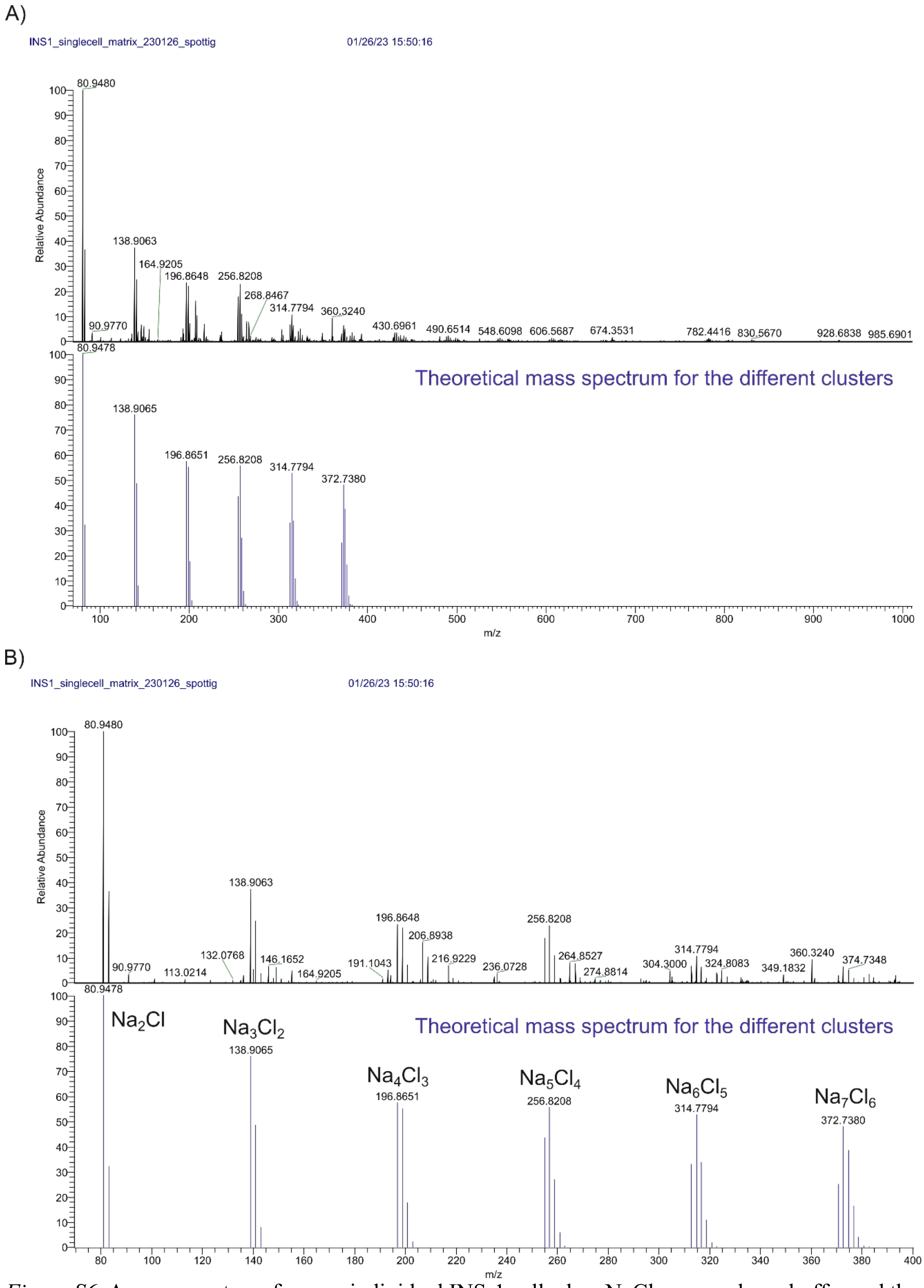

Figure S6. A mass spectrum from an individual INS-1 cell when NaCl was used as a buffer and the cell material was extracted with MeOH:H_2O. A) Mass spectrum ranging from *m/z* 70 to 1000. B) Mass spectrum ranging from *m/z* 70 to 400. The 10 spectra are averaged and background subtracted with scans acquired after the cell was analyzed using XCalibur. At the bottom of each figure, there is the theoretical mass spectrum for the different NaCl clusters as predicted by XCalibur software.

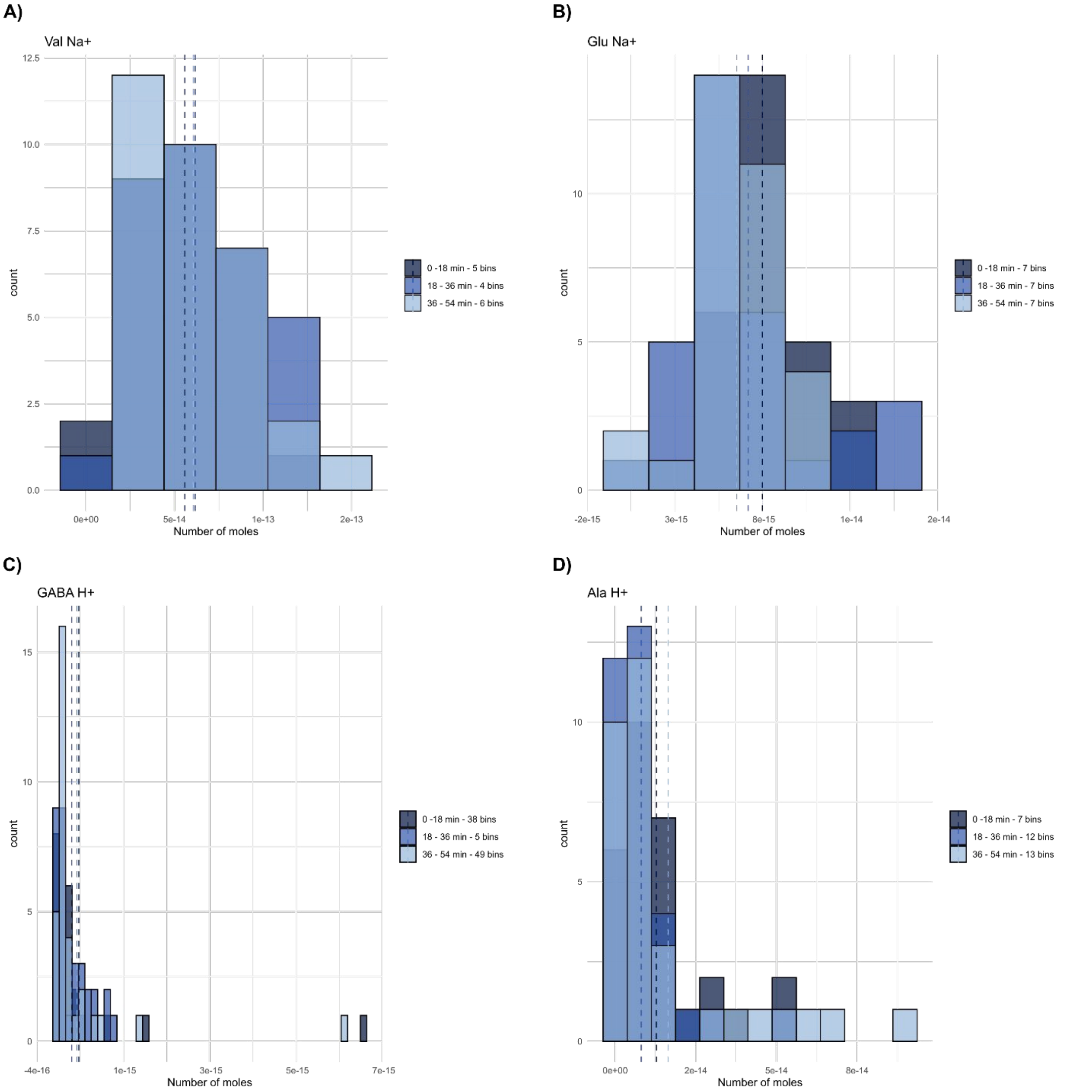

Figure S7. Histograms showing the distribution of the estimated number of moles for different endogenous metabolites detected from INS-1 cells at different time points, namely, 0-18, 18-36, and 36-54 min. *A)* $[Val + Na]^+$. *B)* $[Glu + Na]^+$. *C)* $[GABA + H]^+$. *D)* $[Ala + H]^+$. The number of bins for each treatment group was determined based on the interquartile range. The detected concentration between the groups were not significant according to a two-tailed unpaired heteroscedastic Student's t test analysis, for the exception of the time points 0-18 and 36-54 min $[Glu + Na]^+$. This could be explained by the presence of the value 0 (not detected or quantified) for two cells of the last group.

References

(1) Dimri, G. P.; Lee, X.; Basile, G.; Acosta, M.; Scott, G.; Roskelley, C.; Medrano, E. E.; Linskens, M.; Rubelj, I.; Pereira-Smith, O.; Peacocke, M.; Campisi, J. A Biomarker That Identifies Senescent Human Cells in Culture and in Aging Skin in Vivo. *Proc. Natl. Acad. Sci. U. S. A.* **1995**, *92* (20), 9363–9367. https://doi.org/10.1073/pnas.92.20.9363.

(2) Marques, C.; Liu, L.; Duncan, K. D.; Lanekoff, I. A Direct Infusion Probe for Rapid Metabolomics of Low-Volume Samples. *Anal. Chem.* **2022**, *94* (37), 12875–12883. https://doi.org/10.1021/acs.analchem.2c02918.
